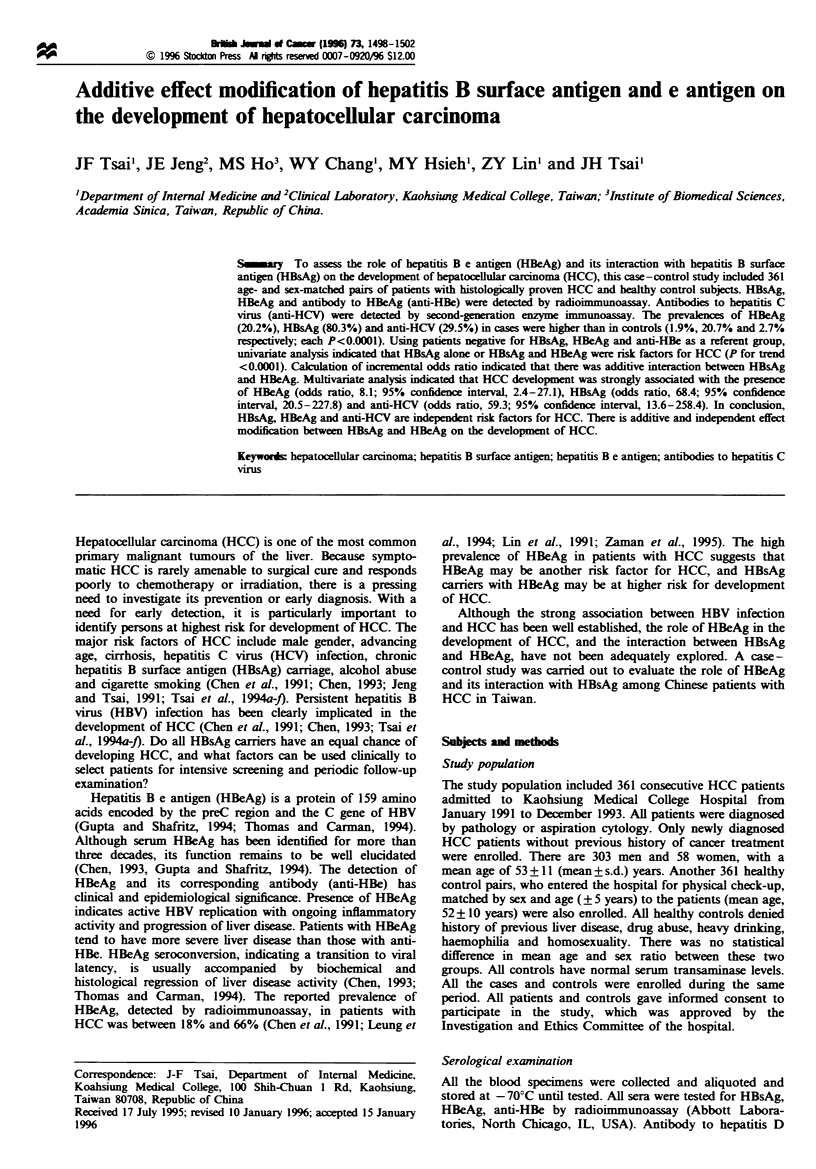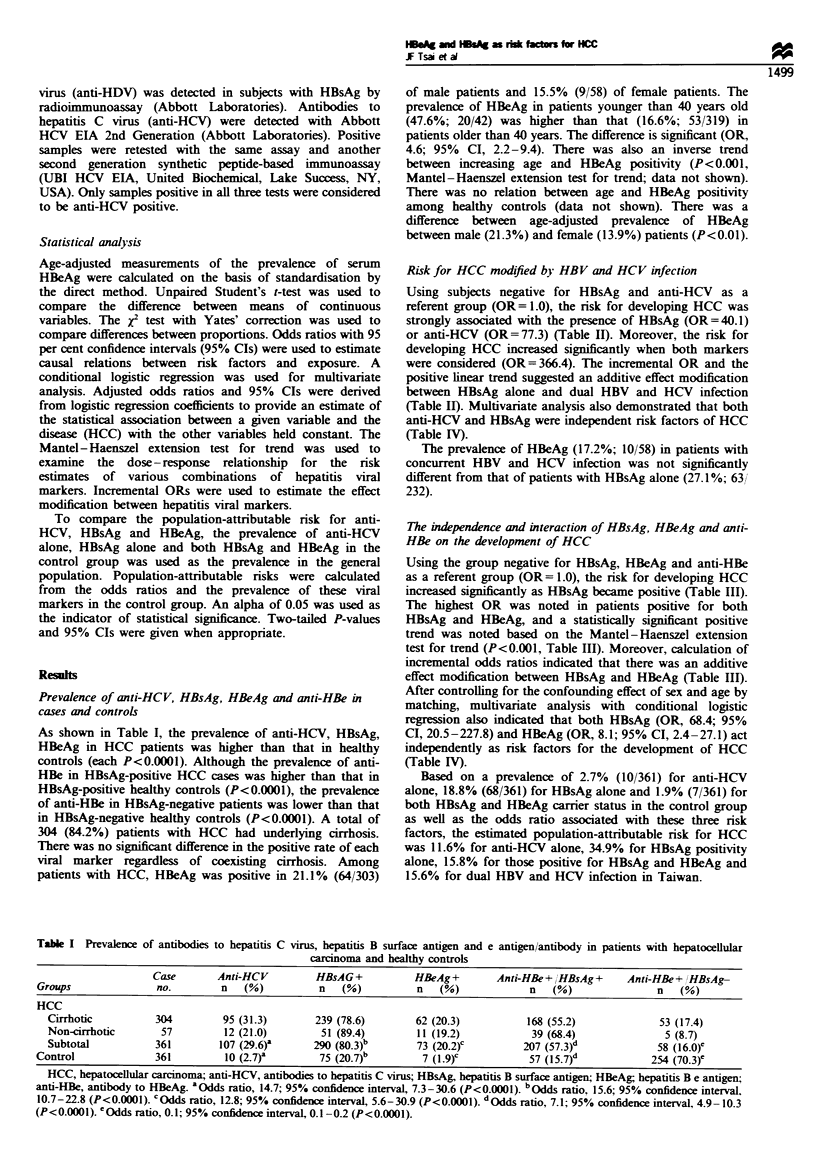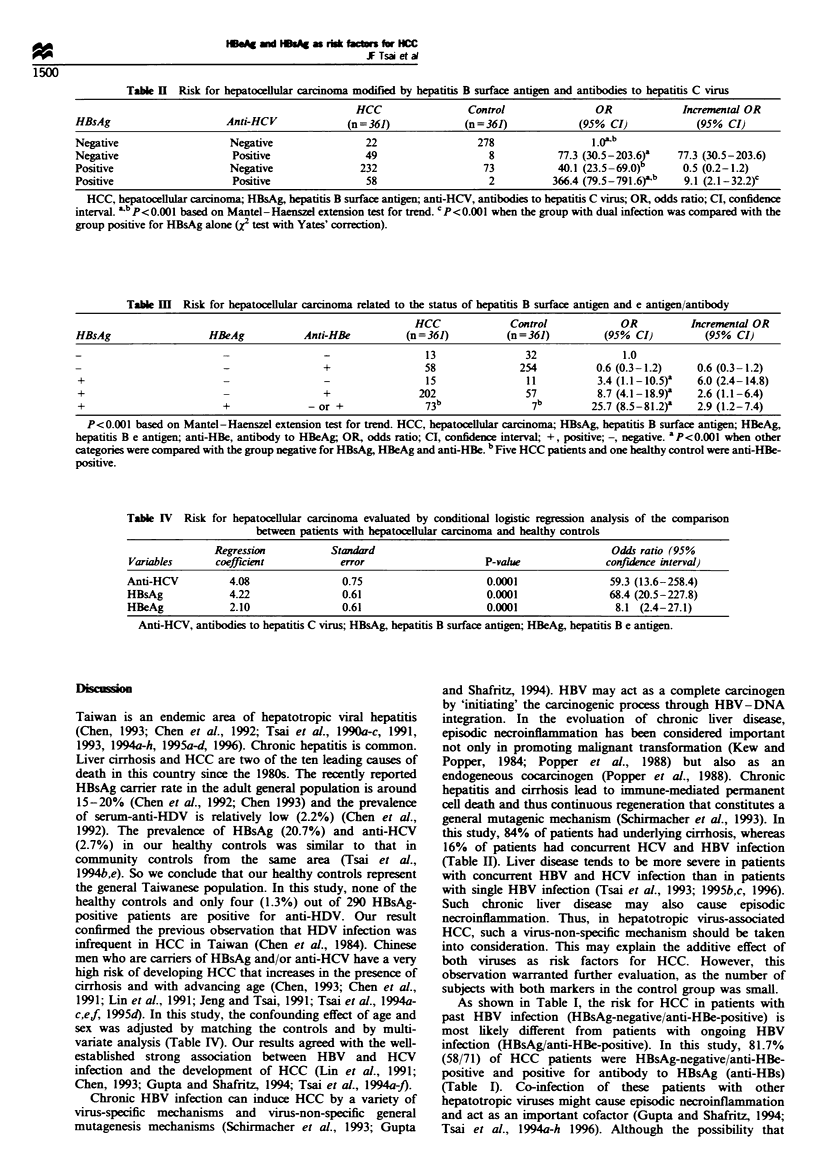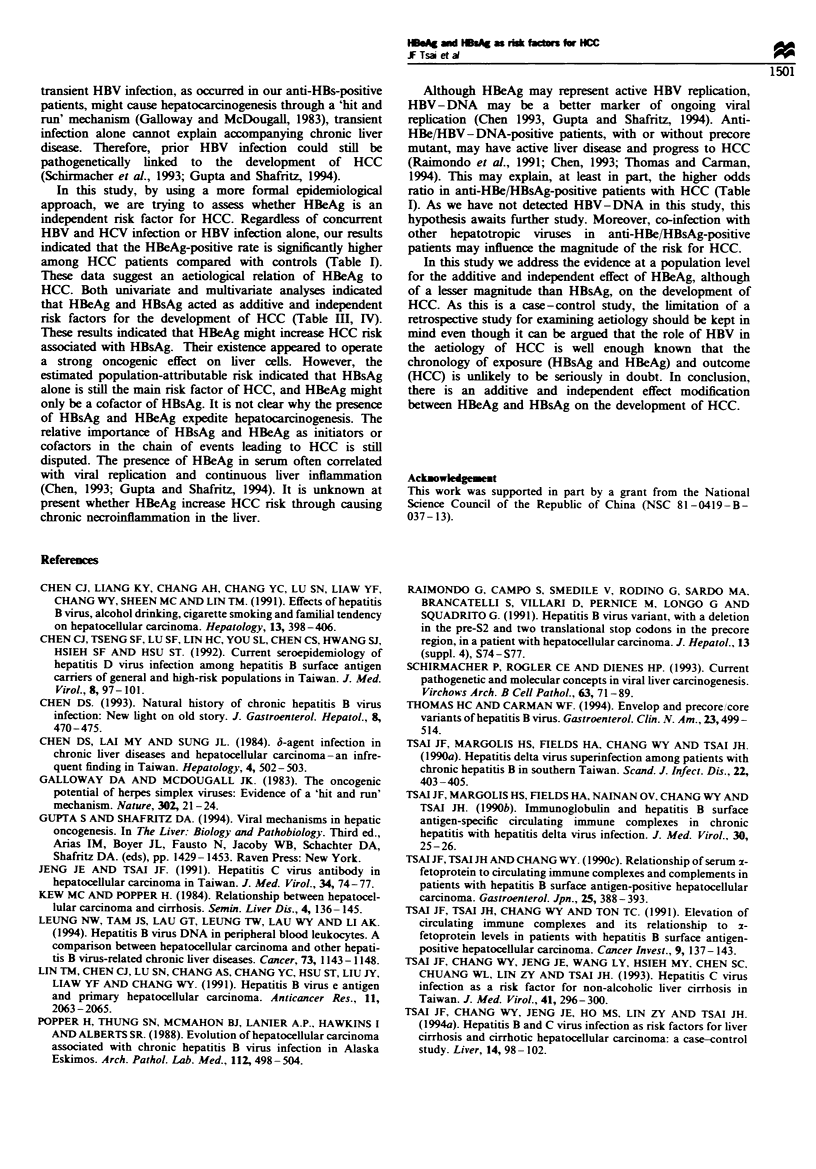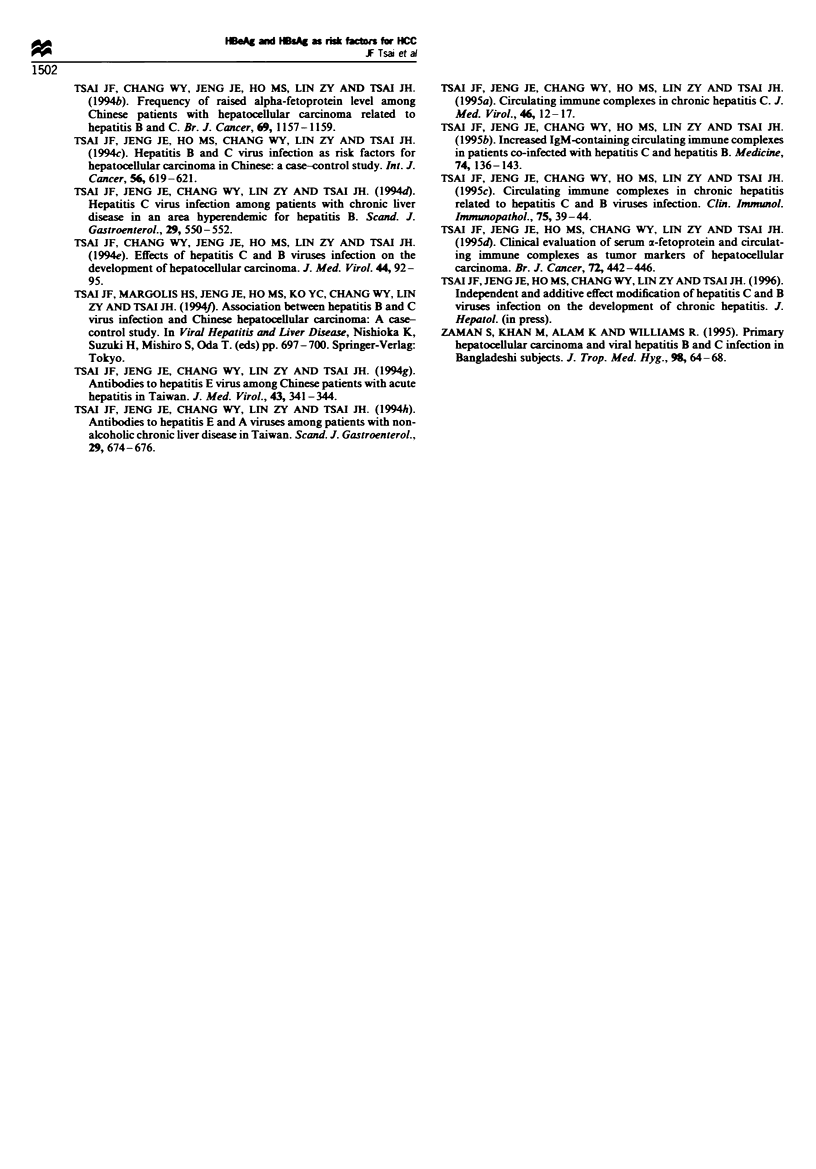# Additive effect modification of hepatitis B surface antigen and e antigen on the development of hepatocellular carcinoma.

**DOI:** 10.1038/bjc.1996.283

**Published:** 1996-06

**Authors:** J. F. Tsai, J. E. Jeng, M. S. Ho, W. Y. Chang, M. Y. Hsieh, Z. Y. Lin, J. H. Tsai

**Affiliations:** Department of Internal Medicine, Kaohsiung Medical College, Taiwan, Republic of China.

## Abstract

To assess the role of hepatitis B e antigen (HBeAg) and its interaction with hepatitis B surface antigen (HBsAg) on the development of hepatocellular carcinoma (HCC), this case-control study included 361 age- and sex-matched pairs of patients with histologically proven HCC and healthy control subjects. HBsAg, HBeAg and antibody to HBeAg (anti-HBe) were detected by radioimmunoassay. Antibodies to hepatitis C virus (anti-HCV) were detected by second-generation enzyme immunoassay. The prevalences of HBeAg (20.2%), HBsAg (80.3%) and anti-HCV (29.5%) in cases were higher than in controls (1.9%, 20.7%, and 2.7% respectively; each P < 0.0001). Using patients negative for HBsAg, HBeAg and anti-HBe as a referent group, univariate analysis indicated that HBsAg alone or HBsAg and HBeAg were risk factors for HCC (P for trend < 0.0001). Calculation of incremental odds ratio indicated that there was additive interaction between HBsAg and HBeAg. Multivariate analysis indicated that HCC development was strongly associated with the presence of HBeAg (odds ratio, 8.1; 95% confidence interval, 2.4-27.1), HBsAg (odds ratio, 68.4; 95% confidence interval, 20.5-227.8) and anti-HCV (odds ratio, 59.3; 95% confidence interval, 13.6-258.4). In conclusion, HBsAg, HBeAg and anti-HCV are independent risk factors for HCC. There is additive and independent effect modification between HBsAg and HBeAg on the development of HCC.


British Journal of Cancer (1996) 73, 1498-1502

© 1996 Stockton Press All rights reserved 0007-0920/96 $12.00

### Additive effect modification of hepatitis B surface antigen and e antigen on the development of hepatocellular carcinoma

JF Tsai[1], JE Jeng[2], MS Ho[3], WY Chang[1], MY Hsieh[1], ZY Lin[1] and JH Tsai[1]

[1]*Department of Internal Medicine and* [2]*Clinical Laboratory, Kaohsiung Medical College, Taiwan;* [3]*Institute of Biomedical Sciences, Academia Sinica, Taiwan, Republic of China.*

**Summary** To assess the role of hepatitis B e antigen (HBeAg) and its interaction with hepatitis B surface antigen (HBsAg) on the development of hepatocellular carcinoma (HCC), this case-control study included 361 age- and sex-matched pairs of patients with histologically proven HCC and healthy control subjects. HBsAg, HBeAg and antibody to HBeAg (anti-HBe) were detected by radioimmunoassay. Antibodies to hepatitis C virus (anti-HCV) were detected by second-generation enzyme immunoassay. The prevalences of HBeAg (20.2%), HBsAg (80.3%) and anti-HCV (29.5%) in cases were higher than in controls (1.9%, 20.7% and 2.7% respectively; each $P<0.0001$). Using patients negative for HBsAg, HBeAg and anti-HBe as a referent group, univariate analysis indicated that HBsAg alone or HBsAg and HBeAg were risk factors for HCC (*P* for trend $<0.0001$). Calculation of incremental odds ratio indicated that there was additive interaction between HBsAg and HBeAg. Multivariate analysis indicated that HCC development was strongly associated with the presence of HBeAg (odds ratio, 8.1; 95% confidence interval, 2.4-27.1), HBsAg (odds ratio, 68.4; 95% confidence interval, 20.5-227.8) and anti-HCV (odds ratio, 59.3; 95% confidence interval, 13.6-258.4). In conclusion, HBsAg, HBeAg and anti-HCV are independent risk factors for HCC. There is additive and independent effect modification between HBsAg and HBeAg on the development of HCC.

**Keywords:** hepatocellular carcinoma; hepatitis B surface antigen; hepatitis B e antigen; antibodies to hepatitis C virus

Hepatocellular carcinoma (HCC) is one of the most common primary malignant tumours of the liver. Because symptomatic HCC is rarely amenable to surgical cure and responds poorly to chemotherapy or irradiation, there is a pressing need to investigate its prevention or early diagnosis. With a need for early detection, it is particularly important to identify persons at highest risk for development of HCC. The major risk factors of HCC include male gender, advancing age, cirrhosis, hepatitis C virus (HCV) infection, chronic hepatitis B surface antigen (HBsAg) carriage, alcohol abuse and cigarette smoking (Chen *et al.*, 1991; Chen, 1993; Jeng and Tsai, 1991; Tsai *et al.*, 1994*a-f*). Persistent hepatitis B virus (HBV) infection has been clearly implicated in the development of HCC (Chen *et al.*, 1991; Chen, 1993; Tsai *et al.*, 1994*a-f*). Do all HBsAg carriers have an equal chance of developing HCC, and what factors can be used clinically to select patients for intensive screening and periodic follow-up examination?

Hepatitis B e antigen (HBeAg) is a protein of 159 amino acids encoded by the preC region and the C gene of HBV (Gupta and Shafritz, 1994; Thomas and Carman, 1994). Although serum HBeAg has been identified for more than three decades, its function remains to be well elucidated (Chen, 1993, Gupta and Shafritz, 1994). The detection of HBeAg and its corresponding antibody (anti-HBe) has clinical and epidemiological significance. Presence of HBeAg indicates active HBV replication with ongoing inflammatory activity and progression of liver disease. Patients with HBeAg tend to have more severe liver disease than those with anti-HBe. HBeAg seroconversion, indicating a transition to viral latency, is usually accompanied by biochemical and histological regression of liver disease activity (Chen, 1993; Thomas and Carman, 1994). The reported prevalence of HBeAg, detected by radioimmunoassay, in patients with HCC was between 18% and 66% (Chen *et al.*, 1991; Leung *et al.*, 1994; Lin *et al.*, 1991; Zaman *et al.*, 1995). The high prevalence of HBeAg in patients with HCC suggests that HBeAg may be another risk factor for HCC, and HBsAg carriers with HBeAg may be at higher risk for development of HCC.

Although the strong association between HBV infection and HCC has been well established, the role of HBeAg in the development of HCC, and the interaction between HBsAg and HBeAg, have not been adequately explored. A case-control study was carried out to evaluate the role of HBeAg and its interaction with HBsAg among Chinese patients with HCC in Taiwan.

### Subjects and methods

### *Study population*

The study population included 361 consecutive HCC patients admitted to Kaohsiung Medical College Hospital from January 1991 to December 1993. All patients were diagnosed by pathology or aspiration cytology. Only newly diagnosed HCC patients without previous history of cancer treatment were enrolled. There are 303 men and 58 women, with a mean age of $53 \pm 11$ (mean $\pm$ s.d.) years. Another 361 healthy control pairs, who entered the hospital for physical check-up, matched by sex and age ($\pm 5$ years) to the patients (mean age, $52 \pm 10$ years) were also enrolled. All healthy controls denied history of previous liver disease, drug abuse, heavy drinking, haemophilia and homosexuality. There was no statistical difference in mean age and sex ratio between these two groups. All controls have normal serum transaminase levels. All the cases and controls were enrolled during the same period. All patients and controls gave informed consent to participate in the study, which was approved by the Investigation and Ethics Committee of the hospital.

### *Serological examination*

All the blood specimens were collected and aliquoted and stored at $-70°C$ until tested. All sera were tested for HBsAg, HBeAg, anti-HBe by radioimmunoassay (Abbott Laboratories, North Chicago, IL, USA). Antibody to hepatitis D

Correspondence: J-F Tsai, Department of Internal Medicine, Koahsiung Medical College, 100 Shih-Chuan 1 Rd, Kaohsiung, Taiwan 80708, Republic of China
Received 17 July 1995; revised 10 January 1996; accepted 15 January 1996

virus (anti-HDV) was detected in subjects with HBsAg by radioimmunoassay (Abbott Laboratories). Antibodies to hepatitis C virus (anti-HCV) were detected with Abbott HCV EIA 2nd Generation (Abbott Laboratories). Positive samples were retested with the same assay and another second generation synthetic peptide-based immunoassay (UBI HCV EIA, United Biochemical, Lake Success, NY, USA). Only samples positive in all three tests were considered to be anti-HCV positive.

### *Statistical analysis*

Age-adjusted measurements of the prevalence of serum HBeAg were calculated on the basis of standardisation by the direct method. Unpaired Student's *t*-test was used to compare the difference between means of continuous variables. The $\chi^2$ test with Yates' correction was used to compare differences between proportions. Odds ratios with 95 per cent confidence intervals (95% CIs) were used to estimate causal relations between risk factors and exposure. A conditional logistic regression was used for multivariate analysis. Adjusted odds ratios and 95% CIs were derived from logistic regression coefficients to provide an estimate of the statistical association between a given variable and the disease (HCC) with the other variables held constant. The Mantel-Haenszel extension test for trend was used to examine the dose-response relationship for the risk estimates of various combinations of hepatitis viral markers. Incremental ORs were used to estimate the effect modification between hepatitis viral markers.

To compare the population-attributable risk for anti-HCV, HBsAg and HBeAg, the prevalence of anti-HCV alone, HBsAg alone and both HBsAg and HBeAg in the control group was used as the prevalence in the general population. Population-attributable risks were calculated from the odds ratios and the prevalence of these viral markers in the control group. An alpha of 0.05 was used as the indicator of statistical significance. Two-tailed *P*-values and 95% CIs were given when appropriate.

### Results

### *Prevalence of anti-HCV, HBsAg, HBeAg and anti-HBe in cases and controls*

As shown in Table I, the prevalence of anti-HCV, HBsAg, HBeAg in HCC patients was higher than that in healthy controls (each $P<0.0001$). Although the prevalence of anti-HBe in HBsAg-positive HCC cases was higher than that in HBsAg-positive healthy controls ($P<0.0001$), the prevalence of anti-HBe in HBsAg-negative patients was lower than that in HBsAg-negative healthy controls ($P<0.0001$). A total of 304 (84.2%) patients with HCC had underlying cirrhosis. There was no significant difference in the positive rate of each viral marker regardless of coexisting cirrhosis. Among patients with HCC, HBeAg was positive in 21.1% (64/303) of male patients and 15.5% (9/58) of female patients. The prevalence of HBeAg in patients younger than 40 years old (47.6%; 20/42) was higher than that (16.6%; 53/319) in patients older than 40 years. The difference is significant (OR, 4.6; 95% CI, 2.2-9.4). There was also an inverse trend between increasing age and HBeAg positivity ($P<0.001$, Mantel-Haenszel extension test for trend; data not shown). There was no relation between age and HBeAg positivity among healthy controls (data not shown). There was a difference between age-adjusted prevalence of HBeAg between male (21.3%) and female (13.9%) patients ($P<0.01$).

### *Risk for HCC modified by HBV and HCV infection*

Using subjects negative for HBsAg and anti-HCV as a referent group (OR = 1.0), the risk for developing HCC was strongly associated with the presence of HBsAg (OR = 40.1) or anti-HCV (OR = 77.3) (Table II). Moreover, the risk for developing HCC increased significantly when both markers were considered (OR = 366.4). The incremental OR and the positive linear trend suggested an additive effect modification between HBsAg alone and dual HBV and HCV infection (Table II). Multivariate analysis also demonstrated that both anti-HCV and HBsAg were independent risk factors of HCC (Table IV).

The prevalence of HBeAg (17.2%; 10/58) in patients with concurrent HBV and HCV infection was not significantly different from that of patients with HBsAg alone (27.1%; 63/232).

### *The independence and interaction of HBsAg, HBeAg and anti-HBe on the development of HCC*

Using the group negative for HBsAg, HBeAg and anti-HBe as a referent group (OR = 1.0), the risk for developing HCC increased significantly as HBsAg became positive (Table III). The highest OR was noted in patients positive for both HBsAg and HBeAg, and a statistically significant positive trend was noted based on the Mantel-Haenszel extension test for trend ($P<0.001$, Table III). Moreover, calculation of incremental odds ratios indicated that there was an additive effect modification between HBsAg and HBeAg (Table III). After controlling for the confounding effect of sex and age by matching, multivariate analysis with conditional logistic regression also indicated that both HBsAg (OR, 68.4; 95% CI, 20.5-227.8) and HBeAg (OR, 8.1; 95% CI, 2.4-27.1) act independently as risk factors for the development of HCC (Table IV).

Based on a prevalence of 2.7% (10/361) for anti-HCV alone, 18.8% (68/361) for HBsAg alone and 1.9% (7/361) for both HBsAg and HBeAg carrier status in the control group as well as the odds ratio associated with these three risk factors, the estimated population-attributable risk for HCC was 11.6% for anti-HCV alone, 34.9% for HBsAg positivity alone, 15.8% for those positive for HBsAg and HBeAg and 15.6% for dual HBV and HCV infection in Taiwan.

**Table I** Prevalence of antibodies to hepatitis C virus, hepatitis B surface antigen and e antigen/antibody in patients with hepatocellular carcinoma and healthy controls

| *Groups* | *Case* *no.* | *Anti-HCV* n (%) | *HBsAG+* n (%) | *HBeAg+* n (%) | *Anti-HBe+/HBsAg+* n (%) | *Anti-HBe+/HBsAg–* n (%) |
|---|---|---|---|---|---|---|
| HCC | | | | | | |
| Cirrhotic | 304 | 95 (31.3) | 239 (78.6) | 62 (20.3) | 168 (55.2) | 53 (17.4) |
| Non-cirrhotic | 57 | 12 (21.0) | 51 (89.4) | 11 (19.2) | 39 (68.4) | 5 (8.7) |
| Subtotal | 361 | 107 (29.6)[a] | 290 (80.3)[b] | 73 (20.2)[c] | 207 (57.3)[d] | 58 (16.0)[e] |
| Control | 361 | 10 (2.7)[a] | 75 (20.7)[b] | 7 (1.9)[c] | 57 (15.7)[d] | 254 (70.3)[e] |

HCC, hepatocellular carcinoma; anti-HCV, antibodies to hepatitis C virus; HBsAg, hepatitis B surface antigen; HBeAg; hepatitis B e antigen; anti-HBe, antibody to HBeAg. [a]Odds ratio, 14.7; 95% confidence interval, 7.3-30.6 ($P<0.0001$). [b]Odds ratio, 15.6; 95% confidence interval, 10.7-22.8 ($P<0.0001$). [c]Odds ratio, 12.8; 95% confidence interval, 5.6-30.9 ($P<0.0001$). [d]Odds ratio, 7.1; 95% confidence interval, 4.9-10.3 ($P<0.0001$). [e]Odds ratio, 0.1; 95% confidence interval, 0.1-0.2 ($P<0.0001$).

Table II Risk for hepatocellular carcinoma modified by hepatitis B surface antigen and antibodies to hepatitis C virus

| *HBsAg* | *Anti-HCV* | *HCC* (n=*361*) | *Control* (n=*361*) | *OR* (*95% CI*) | *Incremental OR* (*95% CI*) |
|---|---|---|---|---|---|
| Negative | Negative | 22 | 278 | 1.0[a,b] | |
| Negative | Positive | 49 | 8 | 77.3 (30.5–203.6)[a] | 77.3 (30.5–203.6) |
| Positive | Negative | 232 | 73 | 40.1 (23.5–69.0)[b] | 0.5 (0.2–1.2) |
| Positive | Positive | 58 | 2 | 366.4 (79.5–791.6)[a,b] | 9.1 (2.1–32.2)[c] |

HCC, hepatocellular carcinoma; HBsAg, hepatitis B surface antigen; anti-HCV, antibodies to hepatitis C virus; OR, odds ratio; CI, confidence interval. [a,b] $P<0.001$ based on Mantel–Haenszel extension test for trend. [c] $P<0.001$ when the group with dual infection was compared with the group positive for HBsAg alone ($\chi^2$ test with Yates' correction).

Table III Risk for hepatocellular carcinoma related to the status of hepatitis B surface antigen and e antigen/antibody

| *HBsAg* | *HBeAg* | *Anti-HBe* | *HCC* (n=*361*) | *Control* (n=*361*) | *OR* (*95% CI*) | *Incremental OR* (*95% CI*) |
|---|---|---|---|---|---|---|
| – | – | – | 13 | 32 | 1.0 | |
| – | – | + | 58 | 254 | 0.6 (0.3–1.2) | 0.6 (0.3–1.2) |
| + | – | – | 15 | 11 | 3.4 (1.1–10.5)[a] | 6.0 (2.4–14.8) |
| + | – | + | 202 | 57 | 8.7 (4.1–18.9)[a] | 2.6 (1.1–6.4) |
| + | + | – or + | 73[b] | 7[b] | 25.7 (8.5–81.2)[a] | 2.9 (1.2–7.4) |

$P<0.001$ based on Mantel–Haenszel extension test for trend. HCC, hepatocellular carcinoma; HBsAg, hepatitis B surface antigen; HBeAg, hepatitis B e antigen; anti-HBe, antibody to HBeAg; OR, odds ratio; CI, confidence interval; +, positive; –, negative. [a] $P<0.001$ when other categories were compared with the group negative for HBsAg, HBeAg and anti-HBe. [b] Five HCC patients and one healthy control were anti-HBe-positive.

Table IV Risk for hepatocellular carcinoma evaluated by conditional logistic regression analysis of the comparison between patients with hepatocellular carcinoma and healthy controls

| *Variables* | *Regression coefficient* | *Standard error* | P-*value* | *Odds ratio (95% confidence interval)* |
|---|---|---|---|---|
| Anti-HCV | 4.08 | 0.75 | 0.0001 | 59.3 (13.6–258.4) |
| HBsAg | 4.22 | 0.61 | 0.0001 | 68.4 (20.5–227.8) |
| HBeAg | 2.10 | 0.61 | 0.0001 | 8.1 (2.4–27.1) |

Anti-HCV, antibodies to hepatitis C virus; HBsAg, hepatitis B surface antigen; HBeAg, hepatitis B e antigen.

### Discussion

Taiwan is an endemic area of hepatotropic viral hepatitis (Chen, 1993; Chen *et al.*, 1992; Tsai *et al.*, 1990*a-c*, 1991, 1993, 1994*a-h*, 1995*a-d*, 1996). Chronic hepatitis is common. Liver cirrhosis and HCC are two of the ten leading causes of death in this country since the 1980s. The recently reported HBsAg carrier rate in the adult general population is around 15–20% (Chen *et al.*, 1992; Chen 1993) and the prevalence of serum-anti-HDV is relatively low (2.2%) (Chen *et al.*, 1992). The prevalence of HBsAg (20.7%) and anti-HCV (2.7%) in our healthy controls was similar to that in community controls from the same area (Tsai *et al.*, 1994*b,e*). So we conclude that our healthy controls represent the general Taiwanese population. In this study, none of the healthy controls and only four (1.3%) out of 290 HBsAg-positive patients are positive for anti-HDV. Our result confirmed the previous observation that HDV infection was infrequent in HCC in Taiwan (Chen *et al.*, 1984). Chinese men who are carriers of HBsAg and/or anti-HCV have a very high risk of developing HCC that increases in the presence of cirrhosis and with advancing age (Chen, 1993; Chen *et al.*, 1991; Lin *et al.*, 1991; Jeng and Tsai, 1991; Tsai *et al.*, 1994*a-c,e,f*, 1995*d*). In this study, the confounding effect of age and sex was adjusted by matching the controls and by multivariate analysis (Table IV). Our results agreed with the well-established strong association between HBV and HCV infection and the development of HCC (Lin *et al.*, 1991; Chen, 1993; Gupta and Shafritz, 1994; Tsai *et al.*, 1994*a-f*).

Chronic HBV infection can induce HCC by a variety of virus-specific mechanisms and virus-non-specific general mutagenesis mechanisms (Schirmacher *et al.*, 1993; Gupta and Shafritz, 1994). HBV may act as a complete carcinogen by 'initiating' the carcinogenic process through HBV–DNA integration. In the evolution of chronic liver disease, episodic necroinflammation has been considered important not only in promoting malignant transformation (Kew and Popper, 1984; Popper *et al.*, 1988) but also as an endogeneous cocarcinogen (Popper *et al.*, 1988). Chronic hepatitis and cirrhosis lead to immune-mediated permanent cell death and thus continuous regeneration that constitutes a general mutagenic mechanism (Schirmacher *et al.*, 1993). In this study, 84% of patients had underlying cirrhosis, whereas 16% of patients had concurrent HCV and HBV infection (Table II). Liver disease tends to be more severe in patients with concurrent HBV and HCV infection than in patients with single HBV infection (Tsai *et al.*, 1993; 1995*b,c*, 1996). Such chronic liver disease may also cause episodic necroinflammation. Thus, in hepatotropic virus-associated HCC, such a virus-non-specific mechanism should be taken into consideration. This may explain the additive effect of both viruses as risk factors for HCC. However, this observation warranted further evaluation, as the number of subjects with both markers in the control group was small.

As shown in Table I, the risk for HCC in patients with past HBV infection (HBsAg-negative/anti-HBe-positive) is most likely different from patients with ongoing HBV infection (HBsAg/anti-HBe-positive). In this study, 81.7% (58/71) of HCC patients were HBsAg-negative/anti-HBe-positive and positive for antibody to HBsAg (anti-HBs) (Table I). Co-infection of these patients with other hepatotropic viruses might cause episodic necroinflammation and act as an important cofactor (Gupta and Shafritz, 1994; Tsai *et al.*, 1994*a-h* 1996). Although the possibility that transient HBV infection, as occurred in our anti-HBs-positive patients, might cause hepatocarcinogenesis through a 'hit and run' mechanism (Galloway and McDougall, 1983), transient infection alone cannot explain accompanying chronic liver disease. Therefore, prior HBV infection could still be pathogenetically linked to the development of HCC (Schirmacher *et al.*, 1993; Gupta and Shafritz, 1994).

In this study, by using a more formal epidemiological approach, we are trying to assess whether HBeAg is an independent risk factor for HCC. Regardless of concurrent HBV and HCV infection or HBV infection alone, our results indicated that the HBeAg-positive rate is significantly higher among HCC patients compared with controls (Table I). These data suggest an aetiological relation of HBeAg to HCC. Both univariate and multivariate analyses indicated that HBeAg and HBsAg acted as additive and independent risk factors for the development of HCC (Table III, IV). These results indicated that HBeAg might increase HCC risk associated with HBsAg. Their existence appeared to operate a strong oncogenic effect on liver cells. However, the estimated population-attributable risk indicated that HBsAg alone is still the main risk factor of HCC, and HBeAg might only be a cofactor of HBsAg. It is not clear why the presence of HBsAg and HBeAg expedite hepatocarcinogenesis. The relative importance of HBsAg and HBeAg as initiators or cofactors in the chain of events leading to HCC is still disputed. The presence of HBeAg in serum often correlated with viral replication and continuous liver inflammation (Chen, 1993; Gupta and Shafritz, 1994). It is unknown at present whether HBeAg increase HCC risk through causing chronic necroinflammation in the liver.

Although HBeAg may represent active HBV replication, HBV-DNA may be a better marker of ongoing viral replication (Chen 1993, Gupta and Shafritz, 1994). Anti-HBe/HBV-DNA-positive patients, with or without precore mutant, may have active liver disease and progress to HCC (Raimondo *et al.*, 1991; Chen, 1993; Thomas and Carman, 1994). This may explain, at least in part, the higher odds ratio in anti-HBe/HBsAg-positive patients with HCC (Table I). As we have not detected HBV-DNA in this study, this hypothesis awaits further study. Moreover, co-infection with other hepatotropic viruses in anti-HBe/HBsAg-positive patients may influence the magnitude of the risk for HCC.

In this study we address the evidence at a population level for the additive and independent effect of HBeAg, although of a lesser magnitude than HBsAg, on the development of HCC. As this is a case-control study, the limitation of a retrospective study for examining aetiology should be kept in mind even though it can be argued that the role of HBV in the aetiology of HCC is well enough known that the chronology of exposure (HBsAg and HBeAg) and outcome (HCC) is unlikely to be seriously in doubt. In conclusion, there is an additive and independent effect modification between HBeAg and HBsAg on the development of HCC.

**Acknowledgement**
This work was supported in part by a grant from the National Science Council of the Republic of China (NSC 81-0419-B-037-13).

### References

CHEN CJ, LIANG KY, CHANG AH, CHANG YC, LU SN, LIAW YF, CHANG WY, SHEEN MC AND LIN TM. (1991). Effects of hepatitis B virus, alcohol drinking, cigarette smoking and familial tendency on hepatocellular carcinoma. *Hepatology*, **13**, 398-406.

CHEN CJ, TSENG SF, LU SF, LIN HC, YOU SL, CHEN CS, HWANG SJ, HSIEH SF AND HSU ST. (1992). Current seroepidemiology of hepatitis D virus infection among hepatitis B surface antigen carriers of general and high-risk populations in Taiwan. *J. Med. Virol.*, **8**, 97-101.

CHEN DS. (1993). Natural history of chronic hepatitis B virus infection: New light on old story. *J. Gastroenterol. Hepatol.*, **8**, 470-475.

CHEN DS, LAI MY AND SUNG JL. (1984). δ-agent infection in chronic liver diseases and hepatocellular carcinoma-an infrequent finding in Taiwan. *Hepatology*, **4**, 502-503.

GALLOWAY DA AND MCDOUGALL JK. (1983). The oncogenic potential of herpes simplex viruses: Evidence of a 'hit and run' mechanism. *Nature*, **302**, 21-24.

GUPTA S AND SHAFRITZ DA. (1994). Viral mechanisms in hepatic oncogenesis. In *The Liver: Biology and Pathobiology*. Third ed., Arias IM, Boyer JL, Fausto N, Jacoby WB, Schachter DA, Shafritz DA. (eds), pp. 1429-1453. Raven Press: New York.

JENG JE AND TSAI JF. (1991). Hepatitis C virus antibody in hepatocellular carcinoma in Taiwan. *J. Med. Virol.*, **34**, 74-77.

KEW MC AND POPPER H. (1984). Relationship between hepatocellular carcinoma and cirrhosis. *Semin. Liver Dis.*, **4**, 136-145.

LEUNG NW, TAM JS, LAU GT, LEUNG TW, LAU WY AND LI AK. (1994). Hepatitis B virus DNA in peripheral blood leukocytes. A comparison between hepatocellular carcinoma and other hepatitis B virus-related chronic liver diseases. *Cancer*, **73**, 1143-1148.

LIN TM, CHEN CJ, LU SN, CHANG AS, CHANG YC, HSU ST, LIU JY, LIAW YF AND CHANG WY. (1991). Hepatitis B virus e antigen and primary hepatocellular carcinoma. *Anticancer Res.*, **11**, 2063-2065.

POPPER H, THUNG SN, MCMAHON BJ, LANIER A.P., HAWKINS I AND ALBERTS SR. (1988). Evolution of hepatocellular carcinoma associated with chronic hepatitis B virus infection in Alaska Eskimos. *Arch. Pathol. Lab. Med.*, **112**, 498-504.

RAIMONDO G, CAMPO S, SMEDILE V, RODINO G, SARDO MA, BRANCATELLI S, VILLARI D, PERNICE M, LONGO G AND SQUADRITO G. (1991). Hepatitis B virus variant, with a deletion in the pre-S2 and two translational stop codons in the precore region, in a patient with hepatocellular carcinoma. *J. Hepatol.*, **13** (suppl. 4), S74-S77.

SCHIRMACHER P, ROGLER CE AND DIENES HP. (1993). Current pathogenetic and molecular concepts in viral liver carcinogenesis. *Virchows Arch. B Cell Pathol.*, **63**, 71-89.

THOMAS HC AND CARMAN WF. (1994). Envelop and precore/core variants of hepatitis B virus. *Gastroenterol. Clin. N. Am.*, **23**, 499-514.

TSAI JF, MARGOLIS HS, FIELDS HA, CHANG WY AND TSAI JH. (1990*a*). Hepatitis delta virus superinfection among patients with chronic hepatitis B in southern Taiwan. *Scand. J. Infect. Dis.*, **22**, 403-405.

TSAI JF, MARGOLIS HS, FIELDS HA, NAINAN OV, CHANG WY AND TSAI JH. (1990*b*). Immunoglobulin and hepatitis B surface antigen-specific circulating immune complexes in chronic hepatitis with hepatitis delta virus infection. *J. Med. Virol.*, **30**, 25-26.

TSAI JF, TSAI JH AND CHANG WY. (1990*c*). Relationship of serum α-fetoprotein to circulating immune complexes and complements in patients with hepatitis B surface antigen-positive hepatocellular carcinoma. *Gastroenterol. Jpn.*, **25**, 388-393.

TSAI JF, TSAI JH, CHANG WY AND TON TC. (1991). Elevation of circulating immune complexes and its relationship to α-fetoprotein levels in patients with hepatitis B surface antigen-positive hepatocellular carcinoma. *Cancer Invest.*, **9**, 137-143.

TSAI JF, CHANG WY, JENG JE, WANG LY, HSIEH MY, CHEN SC, CHUANG WL, LIN ZY AND TSAI JH. (1993). Hepatitis C virus infection as a risk factor for non-alcoholic liver cirrhosis in Taiwan. *J. Med. Virol.*, **41**, 296-300.

TSAI JF, CHANG WY, JENG JE, HO MS, LIN ZY AND TSAI JH. (1994*a*). Hepatitis B and C virus infection as risk factors for liver cirrhosis and cirrhotic hepatocellular carcinoma: a case-control study. *Liver*, **14**, 98-102.

TSAI JF, CHANG WY, JENG JE, HO MS, LIN ZY AND TSAI JH. (1994*b*). Frequency of raised alpha-fetoprotein level among Chinese patients with hepatocellular carcinoma related to hepatitis B and C. *Br. J. Cancer*, **69**, 1157–1159.

TSAI JF, JENG JE, HO MS, CHANG WY, LIN ZY AND TSAI JH. (1994*c*). Hepatitis B and C virus infection as risk factors for hepatocellular carcinoma in Chinese: a case–control study. *Int. J. Cancer*, **56**, 619–621.

TSAI JF, JENG JE, CHANG WY, LIN ZY AND TSAI JH. (1994*d*). Hepatitis C virus infection among patients with chronic liver disease in an area hyperendemic for hepatitis B. *Scand. J. Gastroenterol.*, **29**, 550–552.

TSAI JF, CHANG WY, JENG JE, HO MS, LIN ZY AND TSAI JH. (1994*e*). Effects of hepatitis C and B viruses infection on the development of hepatocellular carcinoma. *J. Med. Virol.* **44**, 92–95.

TSAI JF, MARGOLIS HS, JENG JE, HO MS, KO YC, CHANG WY, LIN ZY AND TSAI JH. (1994*f*). Association between hepatitis B and C virus infection and Chinese hepatocellular carcinoma: A case–control study. In *Viral Hepatitis and Liver Disease*, Nishioka K, Suzuki H, Mishiro S, Oda T. (eds) pp. 697–700. Springer-Verlag: Tokyo.

TSAI JF, JENG JE, CHANG WY, LIN ZY AND TSAI JH. (1994*g*). Antibodies to hepatitis E virus among Chinese patients with acute hepatitis in Taiwan. *J. Med. Virol.*, **43**, 341–344.

TSAI JF, JENG JE, CHANG WY, LIN ZY AND TSAI JH. (1994*h*). Antibodies to hepatitis E and A viruses among patients with non-alcoholic chronic liver disease in Taiwan. *Scand. J. Gastroenterol.*, **29**, 674–676.

TSAI JF, JENG JE, CHANG WY, HO MS, LIN ZY AND TSAI JH. (1995*a*). Circulating immune complexes in chronic hepatitis C. *J. Med. Virol.*, **46**, 12–17.

TSAI JF, JENG JE, CHANG WY, HO MS, LIN ZY AND TSAI JH. (1995*b*). Increased IgM-containing circulating immune complexes in patients co-infected with hepatitis C and hepatitis B. *Medicine*, **74**, 136–143.

TSAI JF, JENG JE, CHANG WY, HO MS, LIN ZY AND TSAI JH. (1995*c*). Circulating immune complexes in chronic hepatitis related to hepatitis C and B viruses infection. *Clin. Immunol. Immunopathol.*, **75**, 39–44.

TSAI JF, JENG JE, HO MS, CHANG WY, LIN ZY AND TSAI JH. (1995*d*). Clinical evaluation of serum α-fetoprotein and circulating immune complexes as tumor markers of hepatocellular carcinoma. *Br. J. Cancer*, **72**, 442–446.

TSAI JF, JENG JE, HO MS, CHANG WY, LIN ZY AND TSAI JH. (1996). Independent and additive effect modification of hepatitis C and B viruses infection on the development of chronic hepatitis. *J. Hepatol.* (in press).

ZAMAN S, KHAN M, ALAM K AND WILLIAMS R. (1995). Primary hepatocellular carcinoma and viral hepatitis B and C infection in Bangladeshi subjects. *J. Trop. Med. Hyg.*, **98**, 64–68.